# Adjustment of Whey:Casein Ratio from 20:80 to 60:40 in Milk Formulation Affects Food Intake and Brainstem and Hypothalamic Neuronal Activation and Gene Expression in Laboratory Mice

**DOI:** 10.3390/foods10030658

**Published:** 2021-03-19

**Authors:** Erin L. Wood, David G. Christian, Mohammed Arafat, Laura K. McColl, Colin G. Prosser, Elizabeth A. Carpenter, Allen S. Levine, Anica Klockars, Pawel K. Olszewski

**Affiliations:** 1 Faculty of Science and Engineering, University of Waikato, Hamilton 3240, New Zealand; erin.lavinia.wood@icloud.com (E.L.W.); dchristian@tutanota.com (D.G.C.); riad_link@yahoo.com (M.A.); Laura.mccoll@waikato.ac.nz (L.K.M.); anica.klockars@waikato.ac.nz (A.K.); 2 Dairy Goat Cooperative, Ltd., Hamilton 3240, New Zealand; Colin.prosser@dgc.co.nz (C.G.P.); Liz.Carpenter@dgc.co.nz (E.A.C.); 3 Department of Food Science and Nutrition, University of Minnesota, St. Paul, MN 55113, USA; aslevine@umn.edu; 4 Department of Integrative Biology and Physiology, Medical School, University of Minnesota, Minneapolis, MN 55414, USA

**Keywords:** brain, satiety, feeding, palatability, milk, formula, animal models

## Abstract

Adjustment of protein content in milk formulations modifies protein and energy levels, ensures amino acid intake and affects satiety. The shift from the natural whey:casein ratio of ~20:80 in animal milk is oftentimes done to reflect the 60:40 ratio of human milk. Studies show that 20:80 versus 60:40 whey:casein milks differently affect glucose metabolism and hormone release; these data parallel animal model findings. It is unknown whether the adjustment from the 20:80 to 60:40 ratio affects appetite and brain processes related to food intake. In this set of studies, we focused on the impact of the 20:80 vs. 60:40 whey:casein content in milk on food intake and feeding-related brain processes in the adult organism. By utilising laboratory mice, we found that the 20:80 whey:casein milk formulation was consumed less avidly and was less preferred than the 60:40 formulation in short-term choice and no-choice feeding paradigms. The relative PCR analyses in the hypothalamus and brain stem revealed that the 20:80 whey:casein milk intake upregulated genes involved in early termination of feeding and in an interplay between reward and satiety, such as melanocortin 3 receptor (MC3R), oxytocin (OXT), proopiomelanocortin (POMC) and glucagon-like peptide-1 receptor (GLP1R). The 20:80 versus 60:40 whey:casein formulation intake differently affected brain neuronal activation (assessed through c-Fos, an immediate-early gene product) in the nucleus of the solitary tract, area postrema, ventromedial hypothalamic nucleus and supraoptic nucleus. We conclude that the shift from the 20:80 to 60:40 whey:casein ratio in milk affects short-term feeding and relevant brain processes.

## 1. Introduction

The nutritional benefits associated with the consumption of milk and dairy products stem from, among others, the macronutrient profile of milk, including the unique protein composition [1,2,3,4]. Milk proteins consist primarily of whey and casein [5]. Unlike the 60:40 whey:casein ratio of human milk, the protein fraction of animal milks (such as bovine and caprine milk predominantly available on the consumer market) has the natural whey:casein ratio of approximately 20:80 [6]; and thus milk formulations used in human nutrition—most commonly infant formulas—are often whey-enhanced to match the 60:40 ratio [7,8,9].

It is well established that proteins, including those present in milk, affect appetite, body weight and metabolic parameters [1,5,10]. Importantly, data showed that whey and casein generate distinct physiological and appetitive responses by interacting with specific transporters and receptors in the gut, affecting nutrient absorption, modifying gastric emptying and gastrointestinal (GI) hormone release [1,5,11,12]. Whey and casein have unique digestion kinetics and post-absorptive effects. Digestion of whey is rapid compared to casein: casein proteins aggregate into curds [13,14], delaying delivery of constituent metabolites to the intestine [11,15,16,17]. Plasma amino acid levels reflect digestion speed, with whey intake inducing higher, immediate increases in circulating amino acids [11,18,19] and casein having delayed and lower but sustained hyperaminoacidemia [11].

Consequently, whey and casein differentially influence the release of some consumption-regulating hormones, which in turn likely produces a unique downstream central nervous system (CNS) response, including activity of relevant brain systems that control appetite. While both fractions produce hypophagia via polypeptide Y (PPY) and its interaction with the Y2 receptor [20,21], whey is a much more potent enhancer of cholecystokinin (CCK), glucagon-like peptide-1 (GLP-1) and gastric inhibitory peptide (GIP) release [16,19,22,23]. As plasma amino acids and gastrointestinal hormones influence feeding either through direct or vagal-mediated action on central pathways [24,25,26,27], scarce prior literature indeed suggests differing effects of whey and casein at central feeding-related circuits, with e.g., whey more effectively modulating serotoninergic activity [28] and expression of select energy homeostasis regulatory genes [29,30,31]. Altered feeding patterns resulting from consumption of either fraction alone have been reported [16,19,32,33].

While evidence delineating the physiological responses to individually presented whey and casein exists, human or laboratory animal studies evaluating the physiological impact of those fractions ingested in milk formulations in two commonly encountered ratios, i.e., whey:casein 60:40 and 20:80, are very scarce. This is surprising considering that these are common ratios in both adult and infant dairy-based nutrition, and one should not simplistically assume that the effect of combined whey and casein in milk formulation would be either negligible or merely “proportional” to their adjusted content. In another study involving healthy adults given a 60:40 versus 20:80 whey:casein milk beverage, a higher whey:casein ratio milk ingested with cereal was found to decrease postprandial glycemia in an insulin-independent manner, primarily through delayed gastric emptying [22]. The authors also observed the preprandial glucose peaks to be lower and GLP-1 plasma levels to be elevated after ingestion of milk with the 60:40 ratio. In line with that, obese rats showed greater improvements in glucose tolerance when fed whey than those given whey plus casein [34,35].

Even though research on whey:casein ratios has been largely spurred by the intent to improve eating behavioral, nutritional and metabolic consequences of protein consumption, surprisingly, very little is known about potential appetite-related feeding and neural consequences that the departure from the natural 20:80 toward 60:40 whey:casein protein ratio in animal milk formulation may produce. This gap in knowledge is particularly critical since it can be presumed that the distinct feeding and neuroendocrine effects shown for whey and casein alone likely contribute to unique appetite-related changes induced by consumption of a formulation containing whey:casein combination at the ratio of 20:80 versus (whey-enhanced) 60:40. Surprisingly, the potential effects of such modification have never been studied. Therefore, the current study utilizing adult laboratory mice was designed to determine whether an adjustment of the whey:casein ratio in protein-matched milk formulation from 20:80 to 60:40 (a) affects palatability and acceptability of the milk formulation in short-term feeding paradigms, (b) whether it evokes a different pattern of activity in feeding-related brain sites after ingestion of the matched amount of one of the formulas, and (c) whether it promotes changes in expression of hypothalamic and brainstem genes critical in food intake regulation. Given the fractions’ differences in post-ingestive effects, we speculate that appetite changes come through central mechanisms uniquely affected by modifications in whey-to-casein content. A standard caprine milk-based formula with the 20:80 versus 60:40 whey:casein ratio was used in the studies.

## 2. Materials and Methods

### 2.1. Animals

Adult male C57Bl mice were individually housed in a temperature-controlled (22 °C) vivarium with a 12:12 h light:dark cycle (with lights on at 09:00). Animals had ad libitum access to standard laboratory chow (Diet 86, Sharpes Stock Feed, Wairarapa, New Zealand) and tap water unless stated otherwise. The procedures were approved by the University of Waikato institutional animal ethics committee (approval #1057).

### 2.2. Milk Formulations

The control formulation contained the natural protein ratio of 20% whey and 80% casein (Control 20:80), whereas the test formulation had 60% whey and 40% casein (60:40). For composition, refer to Table 1. The formulas were goat’s milk-based (Dairy Goat Cooperative, Hamilton, New Zealand), one of the commonly consumed dairy types globally. Both formulations are made using whole (full fat) goat milk, thus both contain goat milk fat. Vegetable oils were added to the formulations to give a comparable fatty acid profile across both formulations. The fat in the 20:80 formulation is 50% milk fat with the rest from vegetable oil (high oleic sunflower, canola and sunflower oil). The 60:40 formulation contains 25% fat from milk and the remainder is vegetable oil (high oleic sunflower, canola, sunflower and coconut oil). They were stored as powder and prepared immediately before use by being reconstituted in water. All animals were pre-exposed to the formulas prior to the trials in order to prevent neophobia.

### 2.3. Feeding Studies

#### 2.3.1. Preference for the Simultaneously Presented Formulations

Mice (*n* = 7–8/group) were acclimatized to two-bottle presentation of the formulations on two separate occasions one week prior to the trial. On the experimental day at 10:00, chow and water were removed from the cages and mice were simultaneously given access to two bottles. One bottle contained the Control 20:80 whey:casein milk formulation and the other, the 60:40 solution. Intake was measured after 2 h by weighing the bottles and the data were expressed in grams.

#### 2.3.2. Energy Deprivation-Induced 2-h Intake of each Formulation Presented Individually Along with Standard Chow

Mice (*n* = 10/group; exposed to the formulations a week earlier on two separate days for 2 h to avoid neophobia) were deprived overnight of chow; water was available during that time. At 10:00, they were given access to standard chow and a bottle containing either the Control 20:80 whey:casein milk formulation or the 60:40 solution for 2 h. Water was removed during the 2-h meal as the formulas were the source of both calories and water. In an additional control scenario, in order to determine the impact of the formulations on consumption of standard chow, another group of mice (*n* = 10) was refed with chow, but instead of either formulation, they received a bottle of water. Chow and fluid consumption was determined at the end of the 2 h meal.

#### 2.3.3. Intake of the Formulations Presented Individually for 24 h

During a 24-h pre-exposure period, to reduce neophobia, a water bottle in each cage was replaced with a bottle containing either the Control 20:80 whey:casein milk formulation or the 60:40 test solution (chow was available). On the experimental day, both chow and water were removed (start at 09:00) and a bottle containing either the Control 20:80 or the 60:40 formulation was placed in the cage. The formulations were the only source of both calories and fluid for the next 24 h. Afterwards, formulation intake was measured in grams.

### 2.4. Neuronal Activation in Feeding-Related Hypothalamic and Brainstem Areas after Consumption of the Same Amount of the Control 60:40 versus 20:80 Whey:Casein Milk Formulation

The presence of an immediate-early gene product, c-Fos, serves as a marker of neuronal activation. The purpose of this experiment was to assess whether consumption of the same amount of the 60:40 whey:casein milk formulation induces a different pattern of neuronal activation in areas of the hypothalamus and brain stem that are crucial in the regulation of food intake compared to the Control 20:80 solution.

Water and standard chow were removed from the cages and animals were presented with either Control 20:80 or the 60:40 whey:casein milk formulation (*n* = 8) for 1 h and allowed to drink 0.35 g (ca. 1 g/g body weight). Mice were then anaesthetized with 35% urethane i.p. and perfused with room-temperature saline (10 mL) and then by 50 mL of ice-cold 4% paraformaldehyde (PFA) solution in 0.1 M phosphate buffer (pH 7.4) one hour after termination of diet exposure. Brains were dissected and postfixed overnight in PFA at 4 °C. Coronal 60 μm vibratome (Leica, Germany) sections were processed for c-Fos immunostaining. c-Fos is a nuclear protein, a product of an intermediate early gene, and its elevated level serves as a marker of neuronal activation. We used a standard c-Fos immunostaining protocol that has been previously employed by our laboratory and by others (for example, [36,37,38]). The Synaptic Systems antibody directed against c-Fos has been previously used by us at the same dilution in rodent free-floating brain sections in immunohistochemical processing [39], and numerous other reports have verified the usability of this primary antibody for mouse and rat brains (e.g., [40,41,42]). The sections were incubated in 3% H2O2 in 10% methanol (in TBS; pH 7.4) for ten minutes, then they were immersed overnight anti-c-Fos antibody (made in rabbit; 1:3000; Synaptic Systems, Göttingen, Germany) at 4 °C. Then sections were incubated for 1 h in the secondary biotinylated goat-anti-rabbit antibody (1:400; Vector Laboratories, Burlingame, CA, USA) and for 1 h in avidin-biotin complex (1:800; Vector Laboratories, Burlingame, CA, USA) at room temperature. 0.05% diaminobenzidine (DAB), 0.01% H_2_O_2_ and 0.2% nickel sulfate (Sigma, St. Louis, MO, USA) were used to visualize cFos-positive nuclei. All incubations were performed in 0.25% gelatin and 0.5% Triton X-100 (Sigma, St. Louis, MO, USA) in TBS. TBS was also used for intermediate rinsing. Sections were mounted onto gelatin-coated slides, air-dried and dehydrated in ethanol (ascending concentrations), placed in xylene and embedded in Entellan (Merck, Darmstadt, Germany). Counting of c-Fos nuclei was performed bilaterally in the regions of interest (4–5 sections/brain) by an investigator blinded to group allocations. Densities of c-Fos immunoreactive nuclei/mm^2^ were averaged per experimental group.

### 2.5. Hypothalamic and Brainstem Gene Expression Following 24-h Exposure to the Control 20:80 versus 60:40 Whey:Casein Milk Formulation

Upon completion of the 24-h Control 20:80 or 60:40 whey:casein milk formulation exposure (as described in Section 2.3.3), the animals were sacrificed at 09:00 by cervical dislocation. The brain stem and hypothalamus were scalpel-dissected from 1 mm thick sections sliced on an ice-cold coronal matrix and the boundaries were determined based on the Allen Brain Atlas (https://mouse.brain-map.org/static/atlas (accessed on 15 January 2020)) reference atlas of the mouse brain. The dissected tissue was immediately placed in Eppendorf tubes containing 1 mL RNALater (Invitrogen, Waltham, MA, USA). The RNALater-immersed hypothalami and brain stems were kept at room temperature for 1 h and then at −80 °C until processing.

Throughout the extraction protocol, the vials were kept on ice with the exception of brief periods of homogenization and centrifuge steps (which were done at 4 °C). Upon thawing, the tissue was transferred from RNALater to TRIzol (Life Technologies, Grand Island, NY, USA; 1 mL/100 mg tissue) and mechanically homogenized. Chloroform (0.2 mL/100 mg tissue) was added and tubes were centrifuged at 4 °C for 20 min at 10,000× *g*. The clear phase with RNA was siphoned, 0.5 mL ice-cold isopropanol was added and samples were put on ice for 10 min. Samples were centrifuged again at 4 °C for 20 min at 10,000× *g*. The aqueous phase was removed and the pellets were resuspended in 0.3 mL ethanol and centrifuged at 4 °C for 10 min at 10,000× *g*. Ethanol was removed, and the pellets were air-dried at room temperature.

Eight µL of DEPC H2O and 1 µL of DNAse buffer (dNature, Gisborne, New Zealand) were added to the pellets. These were incubated with 1 µL DNAse (dNature, Gisborne, New Zealand) at 37 °C for 30 min. DNAse was then inactivated with 1 µL of the stop buffer (dNature, New Zealand) through incubation at 67 °C for 10 min. Removal of genomic DNA was confirmed via PCR using HOT FIREPol Blend Master Mix (dNature, Gisborne, New Zealand), then agarose gel electrophoresis. RNA concentrations were determined with a nanodrop.

cDNA was synthesized from RNA with the iScript Advanced cDNA kit (BioRad, Auckland, New Zealand), confirmed with PCR followed by electrophoresis on the agarose gel.

RT-qPCR determined relative expression of housekeeping genes (ActB, β-tubulin, H3B) as well as of the functional genes of interest. Each reaction consisted of 4 µL of 25 ng/μL sample cDNA, forward and reverse primers (1 µL, 5 µM each), 10 µL iTaq Universal SYBR Green Supermix (BioRad, Auckland, New Zealand) and 4 µL MilliQ water. Reactions were conducted in duplicates with MilliQ water negative controls for each primer pair. Amplification protocol was initiated at 95 °C for 15 min, followed by 45 cycles of 15 s at 95 °C, 15 s at the annealing temperature specific for a primer and 30 s at 72 °C. The following primers were utilized:

ACTB

F: 5′-AGTGTGACGTTGACATCCGT-3′, R: 5′-TGCTAGGAGCCAGAGCAGTA-3′;

BTUB

F: 5′-CGGAAGGAGGCGGAGAGC-3′, R: 5′-AGGGTGCCCATGCCAGAGC-3′;

H3B

F: 5′-CCTTGTGGGTCTGTTTGA-3′, R: 5′-CAGTTGGATGTCCTTGGG-3′;

MC4R

F: 5′-CTTATGATGATCCCAACCCG-3′, R: 5′-GTAGCTCCTTGCTTGCATCC-3′;

POMC

F: 5′-GACACTGGCTGCTCTCCAG-3′, R: 5′-AGCAGCCTCCCGAGACA-3′;

NPY

F: 5′-GGTCTTCAAGCCGAGTTCTG-3′, R: 5′-AACCTCATCACCAGGCAGAG-3′;

KOR

F: 5′-CACCTTGCTGATCCCAAAC-3′, R: 5′-TTCCCAAGTCACCGTCAG-3′;

MOR

F: 5′-CCTGCCGCTCTTCTCTGG-3′. R: 5′-CGGACTCGGTAGGCTGTAAC-3′;

PDYN

F: 5′-GACAGGAGAGGAAGCAGA-3′, R: 5′-AGCAGCACACAAGTCACC-3′;

OXT

F: 5′-CCTACAGCGGATCTCAGACTG-3′, R:5′-TCAGAGCCAGTAAGCCAAGCA-3;

ORX

F: 5′-GCCGTCTCTACGAACTGTTGC-3′, R: 5′-CGCTTTCCCAGAGTCAGGATA-3′;

PNOC

F: 5′-AGCACCTGAAGAGAATGCCG-3′, R: 5′-CATCTCGCACTTGCACCAAG-3′;

OPRL1

F: 5′-ATGACTAGGCGTGGACCTGC-3′, R: 5′-GATGGGCTCTGTGGACTGACA-3′.

### 2.6. Statistical Analyses

Food intake and immunohistochemistry data were analyzed with unpaired Student’s *t*-test for two-group comparisons. In the case of the feeding study where three groups were compared with each other, a one-way ANOVA followed by Tukey’s post hoc test with a correction for multiple comparisons was used. qPCR data analyses were performed with BioRad CX Manager (BioRad, Auckland, New Zealand), followed by unpaired Student’s *t*-test. Differences were deemed statistically significant for *p* < 0.05.

## 3. Results

### 3.1. Feeding Studies

**Figure 1:**
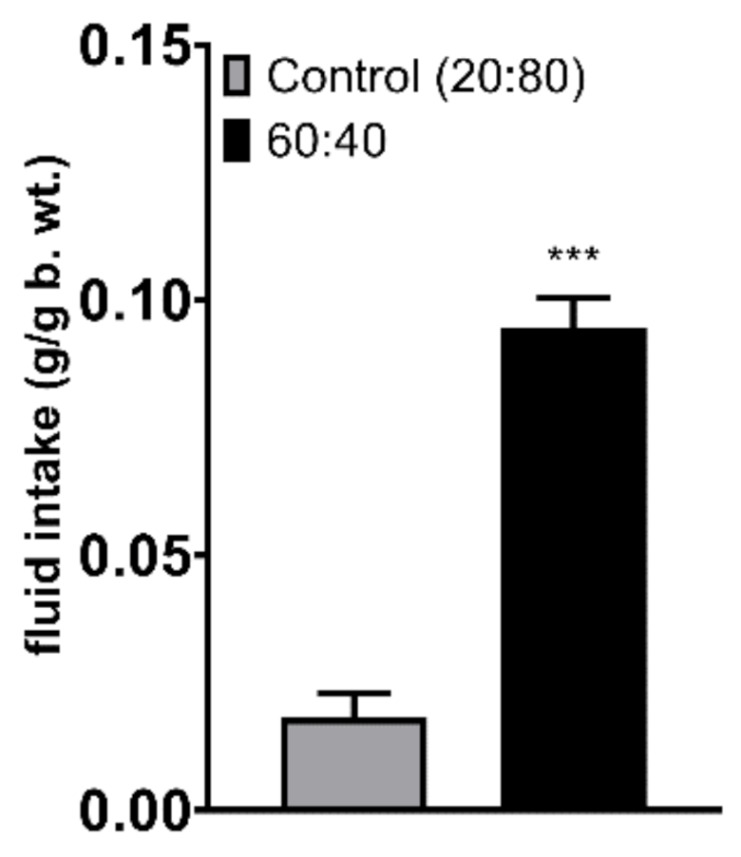
Consumption of the Control 20:80 and 60:40 whey:casein formulation in non-deprived mice during a 2-h episodic exposure of simultaneously presented diets. *** *p* ≤ 0.001.

**Figure 2:**
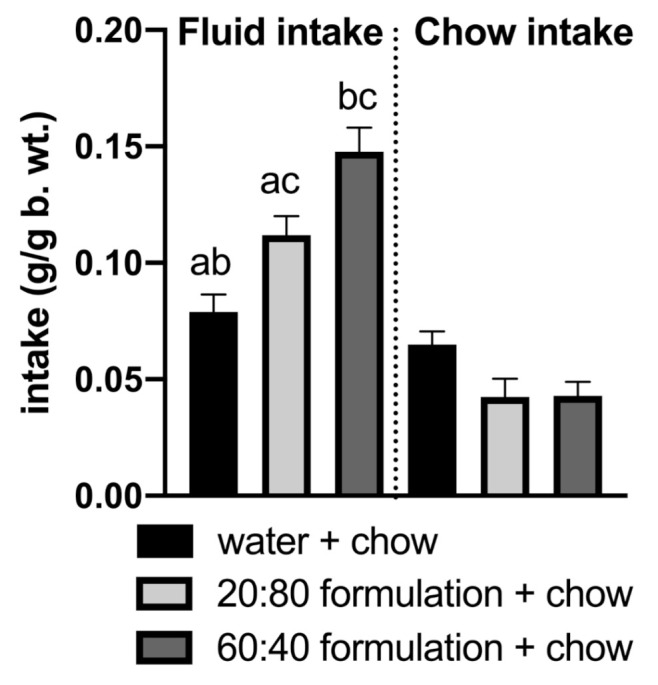
Mice overnight-deprived of food and refed for 2 h with Control 20:80 whey:casein formula + chow or with the 60:40 whey:casein formula + chow or with water + chow most avidly ingested the 60:40 formula followed by the Control 20:80 solution and water. a—significantly different from the 60:40 control formula intake; b—significantly different from the 20:80 formula intake; c—significantly different from the water intake. Significant when *p* < 0.05.

**Figure 3:**
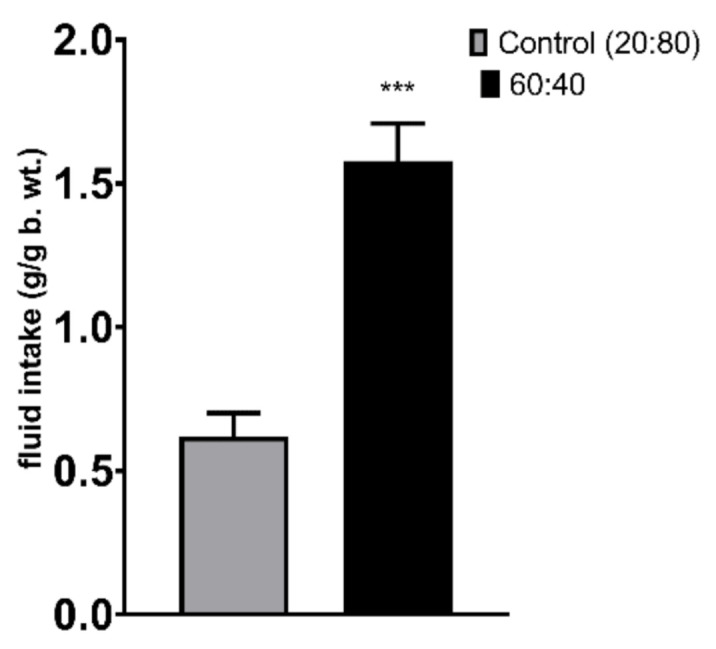
24-h intake of individually presented Control 20:80 and 60:40 whey:casein formulation. *** *p* ≤ 0.001.

During a 2-h two-bottle test in which the animals had a choice between the Control 20:80 formulation and the 60:40 solution, mice showed a significantly lower preference for the Control 20:80 formulation (Figure 1, *p* < 0.001). When the formulas were given independently (no choice between the formulas) along with the standard chow for 2 h to overnight-deprived mice, the animals that had access to the 60:40 solution drank more than the mice given the Control 20:80 formulation (Figure 2, *p* = 0.019). Chow intake did not differ between the two groups. Importantly, the comparison with the group that received water instead of a milk formulation revealed that both formulations were preferred over water (F(2,27) = 3.779); water vs. Control 20:80–*p* = 0.034; water vs. 60:40–*p* < 0.001). Chow intake was lower in the milk groups than in water-given mice, showing a strong trend approaching significance (water vs. Control 20:80–*p* = 0.058; water vs. 60:40–*p* = 0.065; Figure 2). Finally, in the 24-h no-choice exposure to the Control 20:80 versus 60:40 formulations, mice drank less of the Control 20:80 solution (Figure 3, *p* < 0.001).

### 3.2. c-Fos Immunoreactivity

**Figure 4:**
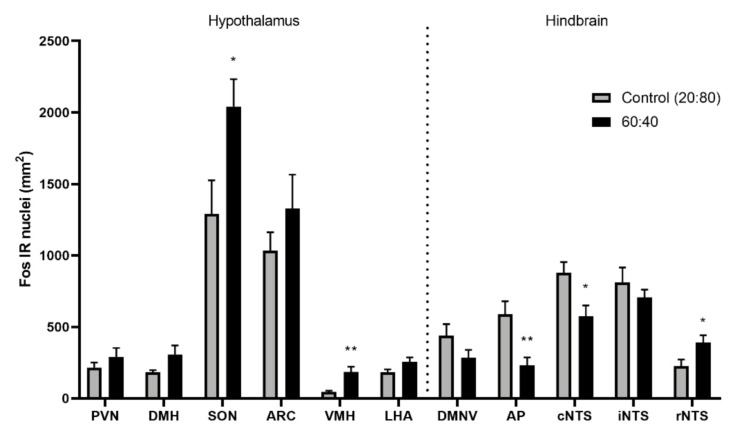
c-Fos immunoreactivity in brain sites related to energy homeostasis increased in the supraoptic nucleus (SON), ventromedial hypothalamus (VMH) and rostral nucleus of the solitary tract (rNTS) and decreased in the area postrema (AP) and caudal nucleus of the solitary tract (cNTS) following the intake of the 60:40 versus control 20:80 whey:casein formulation in mice that ingested 0.35 g (ca. 1 g/g body weight) of the fluid during a 1-h session. PVN–paraventricular nucleus; DMH–dorsomedial hypothalamic nucleus; ARC–arcuate nucleus; LHA–lateral hypothalamic area; DMNV–dorsal motor nucleus of the vagus; iNTS–intermediate nucleus of the solitary tract; * *p* ≤ 0.05; ** *p* ≤ 0.01.

**Figure 5:**
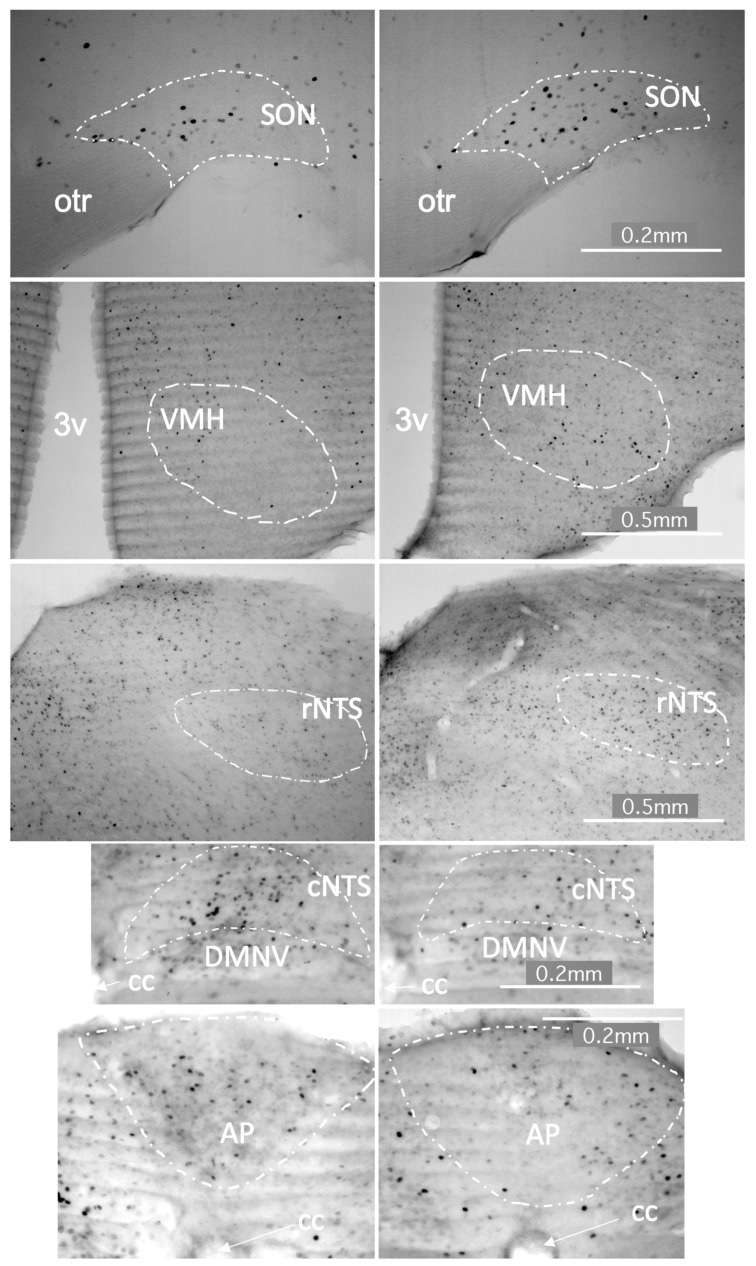
Photomicrographs depicting feeding-related brain sites in which c-Fos immunoreactivity was different after ingestion of the same volume of the 20:80 (**left panel**) vs. 60:40 (**right panel**) whey:casein milk formulation. 3v–third ventricle, AP–area postrema, cc–central canal, cNTS–caudal nucleus of the solitary tract, DMNV–dorsomedial nucleus of the vagus, otr–optic tract, rNTS–rostral nucleus of the solitary tract, SON–supraoptic nucleus, VMH–ventromedial hypothalamic nucleus. Scale bar–0.2 mm (for photographs depicting the SON, cNTS and AP) and 0.5 mm (for VMH and rNTS photographs).

A decrease in c-Fos immunoreactivity was observed in the hypothalamic supraoptic nucleus (SON; *p* = 0.025) as well as the ventromedial hypothalamus (VMH; *p* = 0.008) and the rostral nucleus of the solitary tract (rNTS; *p* = 0.0308) after 1-h exposure to the Control 20:80 whey:casein formulation compared to the 60:40 diet (Figure 4 and Figure 5). Increase in c-Fos IR was noted in the area postrema (AP, *p* = 0.0066) and the caudal portion of the nucleus of the solitary tract (cNTS; *p* = 0.0165).

### 3.3. Gene Expression

**Figure 6:**
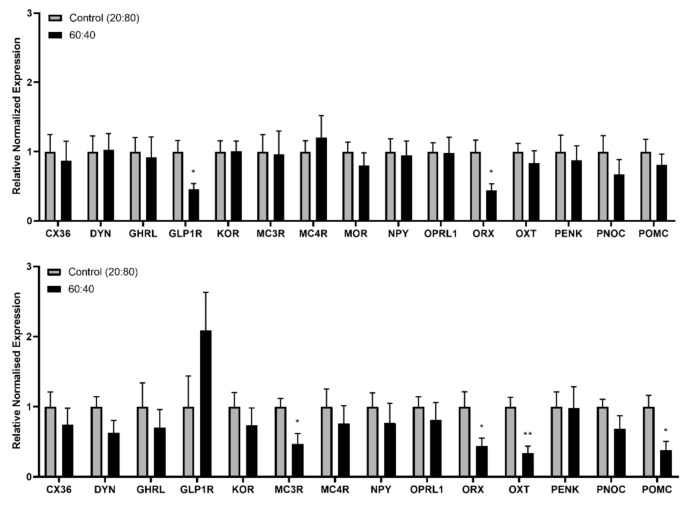
Relative expression of feeding-related genes in the hypothalamus (**top**) and brain stem (**bottom**) after 24-h consumption of the control 20:80 whey:casein formulation versus the 60:40 whey:casein test solution. * *p* ≤ 0.05; ** *p* ≤ 0.01.

Real-time PCR analyses showed an increase in brainstem relative expression of the melanocortin receptor 3 (MC3R; *p* = 0.03), orexin (ORX; *p* = 0.028), oxytocin (OXT; *p* = 0.003) and pro-opiomelanocortin (POMC; *p* = 0.014) genes following consumption of the Control 20:80 formulation compared to the 60:40 solution. Increased expression of glucagon-like peptide-1 receptor (GLP-1R; *p* = 0.033) and ORX (*p* = 0.027) in the hypothalamus was also found with exposure to the Control 20:80 formulation (Figure 6).

## 4. Discussion

While adequate protein intake ensures availability of amino acids, especially the essential ones, and thus supports basic functioning of the organism, excessive protein load may promote adverse effects, such as acidosis or hypercalciuria potentially resulting in kidney disease [43]. It is not surprising, therefore, that intake of protein is a highly regulated process. On the one hand, hunger increases a drive to seek all macronutrients, including protein. However, ingestion of high-protein food triggers early termination of consumption by promoting rapid satiation; and diets very high in protein are perceived as less palatable and their acceptability is relatively low [44,45]. Although the phenomenon of protein intake control has been well described in human and laboratory animal studies, surprisingly little is known about the impact of modifications in protein fraction ratios on appetite. This lack of information is particularly critical in the context of adjusting whey:casein ratios from 20:80 content (i.e., closely resembling animal milks predominant on the consumer market, such as bovine and caprine) to 60:40 in milk formulations.

The current study shows for the first time that a shift from the 20:80 to 60:40 whey:casein ratio in a formulation affects short-term consumption. Also, intake of the 60:40 whey:casein milk produces a different neuronal activation pattern in feeding-related brain areas and a different expression of genes regulating food consumption in the hypothalamus and brain stem than does the 20:80 whey:casein standard formulation.

Notably, we observed in both the 2-h and 24-h exposure paradigms that regardless of the presence of other tastants, the 60:40 whey:casein formulation was consumed in larger quantities and it was preferred over the 20:80 ratio. This consistent outcome across the paradigms employed in our basic research project serves as compelling evidence in that a shift from the 20:80 to 60:40 whey:casein ratio influences acceptability of and preference for milk formula in the short-term. Additional studies are needed in order to elucidate whether this elevated intake of the whey-enhanced milk observed in the brief feeding scenarios persists over a longer time period, especially when this milk is only one of the many components of a more complex and diverse diet. Should this effect indeed persist, thereby leading to a sustained increase in calorie intake, it might translate to long-term consequences for energy homeostasis and body weight. Structural differences between whey and casein and, thus, disparate digestive and postabsorptive responses they evoke, to some extent explain how a change in the proportion of these two fractions contributes to feeding. Casein micelles coalesce in the stomach and form a curd, whereas whey passes through the stomach intact [13,14]. The relative speed of whey digestion is reflected in absorptive processes where more rapid availability of amino acids increases rate of uptake. Whey produces rapid transient peaks in plasma amino acid content, whereas delayed gastric emptying of caseins produces a slower but prolonged elevation of amino acids [11,18,19]. Whey-enhanced formulations are more susceptible to heat-induced protein glycation [46,47] that may reduce their digestibility [48] and perturb the gut bacteria [49]. It is therefore not surprising that whey and casein affect the release of hormones that promote termination of food consumption [24,29,31,32,33,46]. This has been shown, for example, in experimental work focused on an anorexigen, PYY and serotonin. Blood levels of PYY rise at the time of completion of a meal, a baseline PYY concentration during fasting is lower in individuals with obesity, and peripheral injections of this hormone promote a marked reduction in the amount of consumed food [50]. Addition of whey to diets fed to obese rats was found to impact PYY mRNA expression and secretion as well as feeding; and the reduction in food intake was reversed by PYY receptor-2 antagonists. Changes in brain activity have been noted in serotonergic (brain serotonin inhibits food intake, whereas serotonin depletion generates overeating and weight gain [51]) and energy regulating pathways [49,52,53,54,55], though the latter ensues after long-term feeding.

In the context of our general understanding of whey and casein influence on feeding-related mechanisms, the data obtained here further support the notion that while each of the protein fractions alone specifically alters appetite and appetite-related physiological parameters, actual effects of the combined fractions cannot be simplistically extrapolated as proportional to the mere whey:casein ratio. In fact, generalization of appetitive and metabolic effects of whey and casein may be far from possible unless studied in conjunction with specific ratios and with specific foods in which these fractions are used. Indeed, data obtained in previous reports, in which only one fraction or the other was added to diets or administered as a preload, are confusing and oftentimes contradictory. For example, some authors suggested that whey might be suppressing food intake more effectively than casein [19,32]. On the other hand, Marsset-Baglieri et al. found that a liquid snack of whey or casein alone or in combination was effective in suppressing appetite in overweight subjects compared to a maltodextrin control snack; however, there was no difference in satiation potency between the protein groups [16]. Portier et al. gave adult subjects a cheesy snack containing either casein or whey + casein (66:33) as a meal preload [31]. While the preloads lowered intake at the subsequent meal, no differences were observed between the casein and whey + casein groups.

In order to identify the feeding-related physiological consequences of the departure from the conventional 20:80 to the “whey-enhanced” 60:40 ratio, we examined activation and neuronal activation changes in the hypothalamic and hindbrain circuits relevant to appetite regulation. In the brainstem, c-Fos immunoreactivity was increased in the rostral nucleus of the solitary tract (rNTS) and decreased in the area postrema (AP) and caudal nucleus of the solitary tract (cNTS). Immediate response to the 60:40 whey:casein content appears to incorporate gustatory-related signaling through increased rNTS activation, a region with significant gustatory and sensory input [47]. The rNTS displays enhanced activity following oral delivery of strong flavored tastants, such as sweet sucrose, bitter quinine, or sour citric acid [54,55,56,57]. Additionally, activity in the cNTS suggests a role of visceral input contributing to appetitive behaviors. Vagal efferents terminating in the cNTS and the relative permeability of the blood brain barrier in the brain stem allow the combined visceral sensation and circulating nutrients to modulate activity of broader brain pathways [58,59]. The NTS projects extensively to energy homeostasis-related and appetite regulating regions including the PVN, LHA and DMH [58].

In the hypothalamus, the 20:80 whey:casein formulation intake was associated with reduced neuronal activity in the supraoptic nucleus (SON) and the ventromedial hypothalamus (VMH). The reduced c-Fos in these two areas may at first seem counterintuitive. Classically, the SON has been linked with satiety processing as it is targeted by appetite-suppressing cocaine-amphetamine-regulated-transcript, CCK and GLP-1 and it releases—among others—anorexigenic oxytocin [16,22,60]. However, it should be noted that a greater level of c-Fos immunoreactivity in the SON has been linked with palatable high-sugar diet consumption [61]. Furthermore, oxytocin has been also suggested to be relevant to hedonic feeding and food preferences, particularly in relation to sugar consumption. As for the VMH, neurons in this area are able to sense glucose, with some being excited by an increase in glucose concentration, while others inhibited by it, the phenomenon specific to subdivisions of this hypothalamic region [62]. It is important to note that an increase in the activity of VMH neurons has been observed in rats upon sweet taste receptor stimulation with palatable caloric sucrose and non-caloric saccharin solutions [63]. Therefore, it is possible that the higher c-Fos levels observed in the VMH and SON in animals exposed to the 60:40 formula is a consequence of enhanced palatability of the whey-enhanced formulation.

The relative gene expression analyses in the hypothalamus and brain stem following 24-h exposure to the standard 20:80 versus 60:40 formulation revealed that the 20:80 ratio produced higher mRNA expression levels of anorexigenic genes such as MC3R, OXT and POMC in the brainstem and GLP1R in the hypothalamus. This suggests that consumption of the 20:80 whey:casein formula is associated with changes in expression in the melanocortin, OXT and GLP-1 systems, the key players in ensuring early termination of food intake (and in the case of OXT—a possible interplay between the rewarding and satiating effects of the diets that differ in the whey:casein ratio).

Finally, it should be noted that the gastric digestion of goat milk was found to produce thirty-eight individual peptides with antimicrobial, ACE-inhibitory, antioxidant, immunomodulatory, opioid, or dipeptidyl peptidase-IV inhibitory bioactivity [64]. Of these, thirty-four were derived from the casein proteins, and the other four from b-lactoglobulin, a whey protein. Therefore, it is possible that some of the effects described in the present study are not directly due to the added whey, but the reduction in casein proteins. While the identity of the agents is not known, it does not change the interpretation that adjustment of the casein:whey ratio affects the gut–brain axis.

## 5. Conclusions

Overall, we conclude that, in laboratory animal models, a switch from the 20:80 to 60:40 whey:casein formulation impacts formula acceptance and preference, with the whey-enhanced milk being more avidly consumed. The observed differences in the intake of the 20:80 versus 60:40 whey:casein formulations are associated with unique hypothalamic and brainstem c-Fos immunoreactivity and gene expression levels.

## Figures and Tables

**Table 1:** Nutritional composition of prepared milk formulations per 100 mL.

	kJ	Protein (g)	Whey Protein (%)	Fat (g)	Carbohydrate (Lactose, g)
Control (20:80)	278.1	1.3	20.0	3.5	7.5
60:40	275.5	1.4	60.0	3.5	7.1
